# Case-mix adjustments of patient reported experience measures in general practice: variations between Norwegian municipalities

**DOI:** 10.1080/02813432.2026.2675696

**Published:** 2026-05-23

**Authors:** Katrine Skyrud, Øyvind Bjertnæs, Rebecka Norman

**Affiliations:** Cluster of Health Services Research, Norwegian Institute of Public Health, Oslo, Norway

**Keywords:** Patient experience, primary care, quality improvement, case-mix adjustment, Norway

## Abstract

**Background:**

Understanding patients’ experiences with general practice is essential for improving services, and quality indicators are commonly used for that purpose. When reporting at the municipality level, factors beyond municipal control, such as age and country of birth, may influence results. Case-mix adjustment helps ensure fair comparisons. While several international studies have assessed case-mix adjustment for patient experience measures, few studies have examine the impact of case-mix adjustment at the municipality level.

**Objective:**

To identify relevant municipality-level variables for case-mix adjustment in Norway and measure their impact on aggregated patient experience scores.

**Methods:**

We analysed data from the 2023 national survey on patient experiences with the general practitioner. Linear mixed-effects regression was applied with municipalities as a random effect and patient age, sex, long-term conditions, country of birth, education and household income as fixed effects. Outcomes were municipality-level means for six measures: *Assessment of general practitioner*, *Cooperation*, *Enablement, Accessibility*, *Practice*, and *Continuity*.

**Results:**

Age, birth country and long-term conditions were consistently associated with patient experience measures. Intraclass correlations showed that birth country (4%) and education (2.6%) accounted for the greatest between-municipality variation. Case-mix adjustment most strongly influenced *Accessibility* and *Practice* scores. Age and birth country were the most influential adjustors.

**Conclusion:**

Patient characteristics—particularly age and country of birth—are systematically linked to patient reported experience measures, while sex and education are less important. Case-mix adjustment modestly improves fairness in municipality comparisons, underscoring the need for a selective, evidence-based approach when applying adjustment in quality measurement and research.

## Introduction

Understanding patients’ experiences with general practice is necessary for improving service organisation, and quality indicators are commonly used for that purpose [1]. Patient Reported Experiences Measures (PREMs) are survey-based indicators designed to assess patients’ experiences with healthcare services. They are widely used to support improvements in patient-centered care by informing healthcare providers, policymakers, and researchers. PREMs typically assess aspects such as patients’ experiences with communication, accessibility, coordination and enablement. As highlighted by Doyle et al., patient experience is not only a core dimension of healthcare quality but is also positively associated with clinical effectiveness and patient safety [2].

In Norway, municipalities are responsible for the primary care services, including general practitioners (GPs), and GP practices. Therefore, collecting and analysing PREMs at the municipal level could provide valuable insights into how patients’ experience these services. Such information is useful not only for monitoring developments over time, but also for enabling meaningful comparisons across municipalities, grounded in the patients’ own experiences and perspectives [3]. When reporting results at the municipality level, factors beyond municipal control, such as age and country of birth, can impact the results. Case-mix adjustment may therefore be useful to account for such variables, ensuring fair comparisons across municipalities [4]. Conversely, some contend that performance indicators should not be adjusted for case mix, arguing that such adjustments could weaken the commitment to providing the highest quality care to all patients. However, municipalities with a high share of patients who are difficult to treat or more prone to reporting negative experiences may be unfairly penalized if performance incentives rely on unadjusted measures. Therefore, additional research is needed to better support and guide such decisions, particularly in light of the inconsistent application of case‑mix adjustment and the ongoing debate surrounding its value [5,6]. From previous research, it is evident that older age, better self-reported health, and being born in the country where the study is conducted show the strongest and most consistent associations with higher PREM scores across nearly all aspects [5,7–10]. There is some evidence that other characteristics, such as education, marital status, income, employment status, place of living, and sex are associated with survey responses about health care. However, the results are inconsistent and vary among different aspects and measurements [5,11,12]. Some studies have also found associations between PREM scores and characteristics such as health status, type and number of chronic conditions, as well as number of appointments in the previous year [5,12].

Most studies involving case-mix adjustment in PREMs within primary care include adjustment for different sociodemographic variables such as age, sex, education, race/ethnicity as well as self-reported general health, comparing healthcare providers at different organizational or geographical levels, including community health centers, family practice and countries [11,13–17]. The inclusion of self-reported health as a case-mix adjustment variable varies between studies, and its inclusion is debated, as it partly reflects the quality of healthcare services and therefore should not necessarily be adjusted for. Additionally, most previous studies were conducted in countries (e.g. U.S, England and the Netherlands), with healthcare systems different from those in Norway and the Nordic region. Although, several studies from Sweden examined variation in PREM scores at the practice-level, showing associations with characteristics such as patient mix, socioeconomic conditions, practice size, and organisational factors, these did not measure the impact on practice-level comparisons [17–19].

Little is known about the effects of case-mix adjustment on PREMs at the municipality level in primary care settings. The study from Groenewegen et al. [11] developed an empirical approach to identify patient characteristics as potential case-mix adjusters, using GP or countries as comparison units. In this paper we will follow the same approach to identify relevant municipality-level variables for case-mix adjustment across Norwegian municipalities. The aim of the study was to assess the impact of case-mix adjustment on municipality-level scores and identify which patient characteristics have the largest influence on adjusted results. Findings will inform case-mix adjustment of quality indicators at the municipality level, as basis for fairer comparisons across municipalities by adjusting for patient factors outside providers’ control. We address the following research questions:

RQ1: Which patient socio-demographic background variables are associated with PREM scores in general practice; in statistical terms: do they have a statistically significant main effect?

RQ2: Is the variation in the relationship (i.e. the slope variance) between these patient characteristics and PREMs too large to consider their use as case-mix adjusters?

RQ3: Does the distribution of these potential case-mix adjusters differ between municipalities, and what is the relative importance of each individual case-mix adjuster?

RQ4: What is the overall impact of case-mix adjustment on municipality-level PREM scores?

Using data from a national survey conducted at the municipal level, we first identify which variables should be included as case-mix adjusters. We then quantify the impact of case-mix adjustment, overall and for each of the individual case-mix variables. The approach is a combination of predictive and causal, building on the empirical patient case-mix approach from Groenewegen et al [11]. In our study, we predict municipality level scores given an average distribution on patient case-mix factors, while the identification of case-mix factors is done by using regression-based methods of associations with PREMs.

## Material and methods

We utilized data from a national survey on patient experiences with GPs and GP practices, commissioned by the Norwegian Ministry of Health and Care Services and carried out by the Norwegian Institute of Public Health (NIPH) in Norway during 2023–2024.

### Norwegian context

In Norway, the municipalities (*n* = 356) are responsible for offering primary care services, including GP practice. Every resident is entitled to a regular GP through the regular GP scheme, with approximately 99% of the population participating in it [20]. GP practices are typically organized into small units consisting of 2–5 GPs, the majority of whom are self-employed [21]. GPs serve as gatekeepers to specialized healthcare.

### Sampling and data collection

Patients aged 18 years and older who had at least one GP consultation in the past 12 months were eligible for inclusion. In each of Norway’s 356 municipalities, a random selection of 400 patients meeting the inclusion criteria was made. In municipalities with fewer than 400 eligible patients, all were included. Patients registered on the national health portal Helsenorge.no received a digital survey invitation with up to three SMS reminders. Those who are not registered received a postal invitation with one mail reminder. In both cases, the only response option was digital. More information about the sampling and data collection can be found in the report from this survey [3].

### Data

Data from the Norwegian patient experience with GP survey were analysed (*N* = 137 629). Patients with missing information om place of residence, those who were deceased, or had opt out of participation were excluded. We received responses from 59 193 patients, giving a response rate of 43% across 362 municipalities. For the purpose of anonymisation of GPs in the national report [3], municipalities with fewer than 3 GPs were lumped together to one unit, resulting in 294 units out of the original 362 municipalities.

### Patients experience subscales (outcomes)

The Norwegian patient experience with GP questionnaire (PEQ-GP) consists of five subscales: *Assessment of GP* (8 items), *Enablement* (3 items), *Accessibility* (2 items), and *Practice* (3 items), *Cooperation* (2 items; the latter only administered to patients with at least one long-term condition) [3,22]. The subscales have been developed and validated previously, to capture distinct aspects of patient experience with their GP and GP practice [23]. Subscale scores were calculated by transforming the five-point response scale to a 0–100 scale, and computing the mean of available item scores, provided that the respondent completed at least 50% of the items within the subscale. In addition, we included a single item creating a scale on *Continuity*: ‘Do you normally see your own GP when you have an appointment?’. Response options were coded as followed: ‘Yes’, ‘No – a regular substitute’ = 100), ‘No – different doctors’ and ‘Not applicable’ = 0. The questions and number of responses underlying each scale are presented in Table S1.

### Potential case-mix variables

We investigated six individual patient characteristics as potential case-mix adjusters, based on the literature [11,13,15]. Five were derived from registry data and included the following: *sex* (male, female); *age* in four groups (18-49, 59-66, 67-79 and 80+); *country of birth*, categorized into three groups: 1) Norwegian, 2) Africa, Asia, and South America (abbreviated as AfAsSA), and 3) Europe, North America, and Oceania (abbreviated as ENAO); *education*, divided into three groups, based on the ISCED-2011 classification (1) primary school or lower, 2) high school and 3) university or higher); and *household income*, categorized into three groups based on average income after taxes per consumption unit by income deciles, EU-60 scale (1) Decile 1-2, 2) Decile 3-7 and 3) Decile 8+). The sixth characteristic was obtained from survey data on the *number of self-reported long-term conditions*, categorized as follows: 1) none, 2) one, 3) two, and 4) three or more.

### Analysis

The analyses were divided into two steps. The first step was to identify the relevant case-mix adjusters (RQ1-RQ2). We applied multilevel regression analysis for each of the outcome PREM subscales, using a two-level model with patients nested in municipalities. We used linear mixed effects regression models with municipality as a random effect and case-mix adjusters as fixed effects. We fitted bivariate models including one independent variable at a time to determine whether there was an association between the potential adjusters and the subscales. If no relationship was found, there was no need to adjust for that variable. Following this, random slopes were included for the potential case-mix adjusters. If the slope variation turned out to be statistically significant and large enough, correction for case-mix was not indicated. This may be an indication that some municipalities ‘produce’ more similar outcomes across categories (e.g. sex) than others. The slope variance was considered large enough if the difference in variance between categories was more than 0.25 times the variance in the model with fixed effects and random intercept (using the same criterion as Groenewegen et al.).

The next step was to identify the relative importance of the individual case-mix variables and to measure the impact of case-mix adjustment on municipality-level scores (RQ3-RQ4). To assess the relative importance of individual case-mix adjusters we calculated the average discordance in ranks (across all subscales, and for each individual subscale) using data on Kendall’s Tau. Discordance in ranks under case-mix adjustment, *d*, where *d (d=(1-τ)/2)*, represent the probability that one unit’s higher rank compared to another becomes lower (or vice versa) after case-mix adjustment. We assessed both the contribution of separate individual adjusters (comparing to unadjusted) and the unique contribution of the individual adjuster, comparing a fully adjusted model to a model including all but not the individual variable of interest.

The impact of any case-mix adjuster is a function of two factors: how strongly the adjuster predicts PREM within municipalities, and how much the adjuster varies between municipalities. The intra-class correlation (ICC) is interpretable and useful for random intercepts models. It represents the correlation between two observations within the same cluster. A higher correlation *within* the clusters (i.e. a larger ICC) indicates lower variability *within* clusters and consequently, higher variability *between* clusters.

To quantify the impact of case-mix adjustments of the municipality-level subscale scores, we estimated differences in municipality-level means before and after adjustment using two models. Model 1 included age, sex, and the number of long-term conditions. Model 2 additionally included country of birth, education and household income.

## Results

Descriptives characteristics of the 59,193 respondents are found in Table 1. Fifty-nine per cent of respondents were women; 30% were aged above 70; more than 75% reported having one or more long-term conditions; around 10% were not born in Norway; approximately 35% had university or higher education; and 24% had high household income, defined as above 8 deciles on the EU-scale.

**Table 1.** Characteristics of respondents to the 2023 national survey on patient experience with GP practices.

	Total survey respondents (*n* = 59,193)	% of survey respondents
Sex		
Male	24 479	41.4
Female	34 714	58.6
Age group		
18-49	19 254	32.5
50-69	22 193	37.5
70-79	14 873	25.1
80+	2 873	4.9
Number of self-reported long-term conditions		
None	15 265	25.9
One	19 796	33.6
Two	13 989	23.8
Three or more	9 789	16.6
Birth country		
Norway	53 358	90.1
AfAsSA	1 748	3.0
ENAO	4 013	6.8
Unknown	74	0.001
Education		
Primary school or lower	10 731	18.1
High school	27 842	47.0
University or higher	20 620	34.8
Household income* (EU-scale)		
Decile 1-2	12918	21.8
Decile 3-7	32016	54.1
Decile 8+	14158	23.9
Unknown	101	0.001

* Average income after taxes per consumption unit by income deciles. AfAsSA: Africa, Asia and South Africa. ENAO: Europe, North-America and Oceania.

### Identify relevant case-mix adjusters

The bivariate analysis showed that most of the potential case-mix adjusters were significantly associated with the subscale scores, except for sex for the GP subscale and sex and education for the continuity subscale (Table 2). The ICCs show that birth country and education had the largest between-municipality variation, with 4% and 2.6% of the total variation at the municipality level, respectively (Table 2). The smallest difference was found for sex with only 0.5% at the municipality level. We found no slope effect for any of the potential case-mix adjusters and subscale scores: none of the differences in variance between categories was more than 0.25 times the variance in the models with fixed effects and random intercept.

**Table 2.** Summary of the analyses for the dependent variables, multilevel regression models.

Potential case-mix adjusters	Fixed effect (yes/no)	ICC	Discordance of ranks (%) (Total contribution^a^)	Discordance of ranks (%) (Unique contribution^b^)
**Sex**		0.005	**0.57**	**0.43**
Assessment of GP	no		0.06	0.05
Accessibility	yes		0.10	0.10
Enablement	yes		1.35	1.13
Practice	yes		0.56	0.27
Cooperation	yes		1.25	0.96
Continuity	no		0.08	0.04
**Age group**		0.016	**1.65**	**1.44**
Assessment of GP	yes		0.81	0.86
Accessibility	yes		0.40	0.29
Enablement	yes		1.38	1.55
Practice	yes		3.22	2.60
Cooperation	yes		2.64	2.17
Continuity	yes		1.46	1.18
**Number of self-reported long-term conditions**		0.012	**1.12**	**0.98**
Assessment of GP	yes		1.58	1.35
Accessibility	yes		0.55	0.50
Enablement	yes		2.76	2.43
Practice	yes		0.36	0.30
Cooperation	yes		1.07	1.23
Continuity	yes		0.38	0.10
**Birth country**		0.040	**1.25**	**0.93**
Assessment of GP	yes		1.95	1.77
Accessibility	yes		2.13	2.14
Enablement	yes		0.59	0.23
Practice	yes		1.15	0.58
Cooperation	yes		1.03	0.44
Continuity	yes		0.69	0.41
**Education**		0.026	**0.68**	**0.49**
Assessment of GP	yes		0.87	0.68
Accessibility	yes		1.02	0.89
Enablement	yes		0.64	0.55
Practice	yes		0.40	0.09
Cooperation	yes		0.93	0.66
Continuity	no		0.24	0.07
Household income* (EU-scale)		0.012	**0.73**	**0.44**
Assessment of GP	yes		1.21	0.64
Accessibility	yes		0.68	0.38
Enablement	yes		1.02	0.59
Practice	yes		0.31	0.11
Cooperation	yes		0.93	0.61
Continuity	yes		0.20	0.32

^a^ Total contribution: Assessed by comparing ranks from models adjusting for only the case-mix adjustor being examined to unadjusted ranks.

^b^ Unique contribution: Assessed by comparing ranks from fully adjusted models to ranks from models adjusting for all but the one adjustor being examined. ICC: Intra-class correlation.

Bold numbers are the average across all subscales.

### Relative importance of the individual case-mix adjusters

Table 2 summarises the relative importance of each of the six case-mix adjusters, averaged across all subscales. Age was the most influential adjusters (accounting for about 2% discordance by itself) and sex the least (∼ 0.5% discordance). Birth country (∼1% discordance) and number of long-term conditions (∼1% discordance) also had influential effects. The standardised regression coefficient from the multivariate multilevel models (including only the significant case-mix variables identified in the bivariate analysis) for the different subscales can be found in Table 3. The strongest positive association was observed for patients aged 80 and above on the *Practice* subscale, with a standardised coefficient of 8.4 [95% confidence interval (CI): 7.7–9.1]. On the other hand, the strongest negative association was observed for patients with a birth country from Africa, Asia and South America on the *Accessibility* subscale, with a standardized coefficient of −14.4 [95% CI: −15.7—13.1]. Similar strong negative associations were also found for the *Assessment of GP and Practice* subscales. Additionally strong negative associations were observed across all subscales for patients reporting any long-term conditions. Negative associations were also observed to some extent, for those with low education and household income. Largest variations across municipalities after adjusting for case-mix adjustors were observed for the *Accessibility subscale score* and the *Continuity score* with a municipality variance of 63.1 and 145.5.

**Table 3.** Standardized coefficients and 95% confidence intervals for the case-mix adjustors: age, sex, self-reported long-term condition, country of birth, education and household income, multivariate multilevel linear regression from model M2.

	Assessment of GP Estimate [95% CI)	Accessibility Estimate [95% CI)	Enablement Estimate [95% CI)	Practice Estimate [95% CI)	Cooperation Estimate [95% CI)	Continuity Estimate [95% CI)
Intercept	81.8 [81.2, 82.3]***	66.2 [65.0, 67.3]***	72.2 [71.5, 72.9]***	74.7 [74.0, 75.5]***	71.2 [70.3, 72.0]***	82.8 [81.2, 84.4]***
Male		0.6 [0.1, 1.0]*	2.5 [2.2, 2.9]***	0.8 [0.5, 1.1]***	2.0 [1.5, 2.5]***	
Age group: 50-66	1.9 [1.5, 2.3]***	0.6 [0.1, 1.2]*	2.3 [1.8, 2.7]***	5.7 [5.3, 6.0]***	4.3 [3.7, 4.9]***	3.5 [2.8, 4.2]***
Age group: 67-79	1.4 [1.0, 1.8]***	1.7 [1.1, 2.3]***	3.5 [3.0, 4.0]***	8.0 [7.6, 8.4]***	5.5 [4.9, 6.2]***	5.1 [4.3, 5.8]***
Age group: 80+	−0.3 [−1.0, 0.4]	0.7 [−0.4, 1.8]	2.1 [1.2, 3.1]***	8.4 [7.7, 9.1]***	3.5 [2.4, 4.5]***	3.5 [2.1, 4.8]***
Self-reported long-term condition: One	−1.8 [−2.1, −1.4]***	−1.2 [−1.7, −0.6]***	−4.4 [−4.9, −3.9]***	−0.6 [−0.9, −0.2]**		0.4 [−0.3, 1.1]
Self-reported long-term condition: Two	−2.9 [−3.3, −2.5]***	−2.6 [−3.2, −2.0]***	−6.3 [−6.9, −5.8]***	−1.0 [−1.4, −0.6]***	−1.9 [−2.4, −1.4]***	0.5 [−0.3, 1.3]
Self-reported long-term condition: Three or more	−3.8 [−4.3, −3.3]***	−3.3 [−4.0, −2.6]***	−7.8 [−8.4, −7.2]***	−1.1 [−1.6, −0.7]***	−3.5 [−4.1, −2.9]***	0.8 [−0.1, 1.6] +
Primary school or lower	−1.0 [−1.4, −0.5]***	−3.2 [−3.8, −2.5]***	1.0 [0.4, 1.5]***	−0.1 [−0.5, 0.3]	1.0 [0.4, 1.7]**	
High school	−0.7 [−1.1, −0.4]***	−2.5 [−3.0, −2.0]***	0.7 [0.3, 1.2]***	−0.2 [−0.6, 0.1]	1.0 [0.5, 1.5]***	
Income: Decile 1-2	−1.6 [−2.1, −1.2]***	−2.2 [−2.9, −1.6]***	−1.8 [−2.4, −1.2]***	−0.2 [−0.6, 0.3]	−1.3 [−2.0, −0.6]***	1.5 [0.6, 2.3]***
Income: Decile 3-7	−0.2 [−0.5, 0.2]	−0.8 [−1.4, −0.3]**	0.0 [−0.5, 0.5]	0.2 [−0.1, 0.6]	0.1 [−0.5, 0.6]	1.0 [0.3, 1.6]**
Birth country: AfAsSA	−6.3 [−7.2, −5.4]***	−14.4 [−15.7, −13.1]***	−0.8 [−1.9, 0.3]	−4.2 [−5.0, −3.3]***	−2.0 [−3.4, −0.6]**	−1.8 [−3.5, −0.2]*
Birth country: ENAO	−1.6 [−2.2, −1.0]***	−5.7 [−6.5, −4.8]***	−0.2 [−1.0, 0.5]	0.0 [−0.6, 0.5]	0.2 [−0.7, 1.1]	−1.6 [−2.6, −0.5]**
Num.Obs.	58683	57167	52719	58143	38221	58394
AIC	502363.3	535440.8	473395.8	493570.2	345612.5	575769.7
BIC	502498.0	535584.0	473537.8	493713.8	345740.7	575886.4
Municipality variance	11.3	63.1	11.7	24.2	13.6	145.5
Residual variance	302.5	674.2	460.5	280.5	489.4	1103.3
RMSE	17.35	25.90	21.41	16.71	22.05	33.13

*** *p* < 0.01.

** *p* < 0.05.

* *p* < 0.1. Confidence interval (CI).

Coefficients from model M1 can be found in the Table S2.

### Overall impact of case-mix adjustment

**Figure 1.**
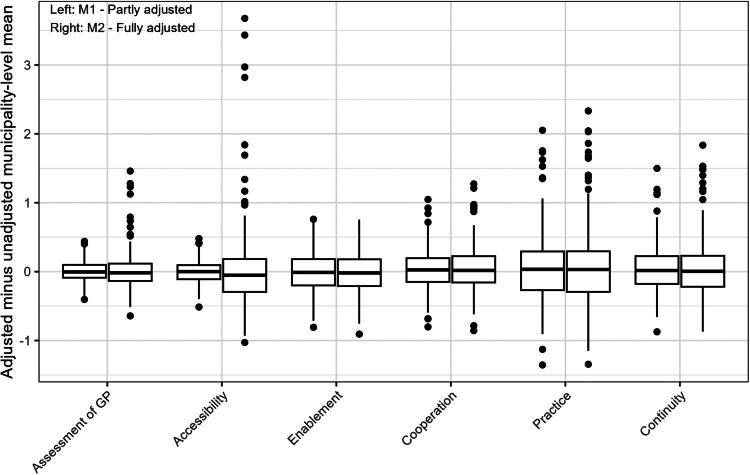
Boxplot showing the impact of case-mix adjustment on municipality mean scale scores (adjusted - unadjusted means). Whiskers show 1.5 IQR beyond the actual IQR. Model 1 (M1) included sex (if significant fixed effect), age and number of long-term conditions, and Model 2 (M2) additionally included birth country, education (if significant fixed effect) and household income.

Figure 1 shows the magnitude and direction of differences in municipality-level mean subscale scores on the six patient experience subscales after case-mix adjustments in Model 1 and Model 2. The proportion of municipalities identified as outliers (more than 1.5 times the IQR outside the actual IQR) ranges from 1.0% to 4.1% across subscales and models. The figure shows that differences in municipality mean subscale scores generally range from 0.5 − 2 points on the 0-100 scale, with larger outlier adjustments being almost entirely positive, that is, representing municipalities whose scores increased as a result of case-mix adjustment. The largest impact on the municipality scores were observed for the *Accessibility* subscale when using M2. A notable effect was also seen for the *Practice* subscale when using M1, but with no further changes when moving to M2.

## Discussion

### Main findings

In this study of 59,193 respondents from 294 municipalities/units, we identified the strongest associations between patient experience scores and age, birth country, and number of long-term conditions, based on linear mixed-effects regression analyses. Birth country and education showed the highest municipality-level mean subscale variation, with ICCs of 4% and 2.6%, respectively. Among the subscales, *Accessibility* was most affected by case-mix adjustment, with age and birth country emerging as the most influential adjustment variables.

### Comparison with existing literature

As expected, significant associations were found between the case-mix variables age, birth country and number of long-term conditions, and PREMs, in line with previous studies [5,8–10,24]. Patients born in Africa, Asia, and South America reported worse experiences across all subscales, with lowest scores for *Accessibility*. Several studies have tried to explain this. A study from U.K found that the negative evaluations may reflect communication issues, and that more negative experiences with waiting times for appointments could reflect differing expectations of care [6]. Similarly, when GPs and patients share the same race or ethnicity, it can lead to better communication, improved patient satisfaction, and greater adherence to treatment plans [25,26]. Our findings on the negative association between low socioeconomic status and PREMs have also been reported in several studies from Sweden [18,19,27]. Another study from Sweden assessed the association between structural and organisational factors and PREMs, both factors with and without provider control [17]. This is outside the scope of the current study, which was restricted to patient case-mix, in line with a common case-mix approach for PREMs and PROMs in primary care [11]. However, more case-mix research including non-patient factors outside provider control is warranted, e.g. the impact of area deprivation on case-mix adjustments [15], as well as research assessing modifiable structural and organisational factors affecting PREMs [17].

Using this newly developed approach to identify patient characteristics as case-mix adjusters, most of the potential case-mix adjusters were significantly associated with the subscales, except for sex for the *Assessment of GP* subscale and sex and education for the *Continuity* subscale. Most other studies also find that patient sex is either not associated with experiences of general practice or has only a small association [7,11,15], including a recent international survey conducted in 19 countries [28]. Accordingly, no significant fixed effects are observed, therefore sex should not be included as a case-mix adjuster. Another reason for not including a variable as case-mix adjuster [11] is if the effect of the potential adjuster varies substantially between units (whether the slope variance is big enough). We used the same criterion [11]: if the difference in variance between categories was more than 0.25 times the variance in the model with fixed effects and random intercept, thus it should not be included as a case-mix adjuster. The rationale is that some providers might perform better across these categories than others, i.e. have more similar outcomes for all categories (e.g. for birth country categories) than others. Hence, outcomes for these categories might be affected by provider actions and case-mix adjustment is not warranted. In contrast to Groenewegen et al. who found important slope effects on both GP and country level, our study did not find any case-mix variable with a large enough difference between the categories. The variance in the model with fixed effects and random intercept is the sum of the municipality-level variance and the individual (residual) variance. A large residual variance, as seen in our study would require a very large difference in the variance between the categories, which were not found in our study.

Similar studies investigating the impact of case-mix adjustments when comparing levels of providers, all state that such adjustment is necessary to account for factors like age and country of birth, ensuring fair comparisons across providers or countries [11,13–15]. However, the impact of case-mix adjustment on practice-level scores varies, from modest impact [9,13] to more substantial impact on the practice scores [15]. Our study identified age and country of birth as the most influential adjusters, but with a smaller effect on municipality-level scores compared to Paddison et al. [15] on the practice level. This is as expected, given that the municipality level is aggregated from underlying practices and GPs and thus both predictors, outcomes and impacts are averaged out across these lower levels. Internationally, the use of case-mix adjustment for PREMs is standard, but there is less agreement on which variables to use in the case-mix model. For example, some studies include self-reported health, others do not. It has been argued that self-reported health is not beyond control of the units, and therefore should not be used as a case-mix adjuster for PREMs [11]. In our study we chose not to include this variable as case-mix adjustment. This is supported by a recent international survey among 19 countries, where 7-15% of the variation on self-reported health measures were at levels above the patient [28].

### Strengths and limitations

A strength of the study is the use of data from a large national survey using a questionnaire developed according to established procedures, and with randomly selected patients within each municipality [3]. However, in some municipalities only a few GPs were working. To ensure anonymity of GPs and increase statistical power, municipalities with fewer than three GPs were lumped together into a single unit. While this approach improves the robustness, it may reduce the geographical specificity of the results and hence, mask potential variation between smaller municipalities. The response rate of 43% is comparable, or higher than in similar large-scale patient experience surveys [15], but we cannot rule out non-response bias. Certain groups of patients, such as individuals with lower digital literacy, lower education, or those with non-Norwegian birth country, may be underrepresented. This could impact the strength of the detectability of associations for some case-mix variables. As a result, the relative importance of certain variables, such as education or income, may have been underestimated in this study. However, a previous follow-up study of non-respondents found only small differences between respondents and non-respondents on patient reported experiences with GPs [23], indicating small consequences of non-response for our study. Another strength of the study is the availiability of rich data, combining registry-based and self-reported measures, which allows for detailed evaluation of potential case-mix adjustment across a broad range of patient characteristics. However, some potentially relevant variables such as language spoken at home and marital status were not availiable. Although these factors could potentially influence the results, they are rarely included in similar studies.

### Implications for research and practice

This study has several implications. First, case-mix adjustment for variables such as age and country of birth make comparisons across municipalities fairer by accounting for population differences. Even small effects can be relevant and important in high-stake applications like public reporting or performance comparisons. For health authorities and policymakers, this highlights the importance of carefully considering case-mix adjustment to avoid unintended bias against municipalities serving more diverse or vulnerable populations, while at the same time also avoiding unnecessary complexity. For research, the study supports a cautious and evidence-based approach to case-mix adjustment of PREMs. It underscores the need to test and justify the inclusion of the adjustment variables and whole model, rather than using a standard set across contexts. Finally, the lack of random slope effects for country of birth at the municipality level does not imply that efforts to address differences between categories are redundant, but rather that all municipalities, GP practices and the policy level should address the poorer scores for persons born in Africa, Asia and South America.

## Conclusion

This study confirms that patient characteristics—particularly age and country of birth—are systematically linked to patient reported experience measures, while other variables such as sex and education have limited influence. The results underscore that case-mix adjustment ensure fairer comparisons between municipalities by accounting for differences in patient populations and is particularly important in high-stake initiatives like public reporting or national policy decisions. At the same time, our findings support a selective approach to case-mix adjustment: not all patient characteristics are relevant, and variables should be included only when there is clear evidence of their influence.

## Supplementary Material

Supplementary.docx

## Data Availability

Anonymous data generated during and/or analysed during the current study are available from the corresponding author on reasonable request.
