# Supplementary material for: Case-mix adjustments of patient reported experience measures in general practice: variations between Norwegian municipalities
Source: Scand J Prim Health Care. 2026 May 23;44(1):2675696. doi: 10.1080/02813432.2026.2675696 (PMC13202693; doi:10.1080/02813432.2026.2675696)
Supplement: Supplementary.docx [file IPRI_A_2675696_SM8279.docx]

# Supplementary materials

**Table S1: The questions underlying each scale**

| **Scales /items ^a^** | **Numbers of response** |
| --- | --- |
| Total sample | **59 193** |
| **Assessment of GP** | **59 077** |
| GP takes you seriously | 58 978 |
| GP spends enough time with you | 58 927 |
| GP talks to you in a way you understand | 58 984 |
| GP is professionally competent | 58 919 |
| GP shows interest in your situation | 58 912 |
| GP includes you as much as you would like in decisions concerning you | 58 865 |
| GP provides sufficient information about health problems and treatment | 58 841 |
| GP provides sufficient information about use/side effects of medication | 43 138 |
| **Practice** | **58 525** |
| GP practice well organized | 57 807 |
| Other employees helpful and competent | 58 216 |
| Treated with courtesy and respect at the reception | 57 848 |
| **Enablement** | **53 050** |
| Contact with GP make you better able to understand your health problems | 53 438 |
| Contact with GP make you better able to cope with your health problems | 52 516 |
| Contact with GP better helps you to stay healthy | 51 749 |
| **Accessibility** | **57 548** |
| Waiting time for your last urgent appointment acceptable | 49 401 |
| Waiting time for appointments that are not urgent acceptable | 55 691 |
| **Continuity of care** | **58 798** |
| Do you normally meet your own doctor | 58 798 |

**Table S2: Standardized coefficients and 95% confidence intervals for the case-mix adjustors: age, sex and self-reported long-term condition, multivariate multilevel linear regression from model M1.**

|  | Assessment of GP  Estimate [95% CI) | Accessibility  Estimate [95% CI) | Enablement  Estimate [95% CI) | Practice  Estimate [95% CI) | Cooperation  Estimate [95% CI) | Continuity  Estimate [95% CI) |
| --- | --- | --- | --- | --- | --- | --- |
| Intercept | 80.3  [79.8, 80.8] *** | 62.3  [61.3, 63.4] *** | 72.1  [71.5, 72.7] *** | 74.5  [73.8, 75.1] *** | 71.3  [70.7, 72.0] *** | 83.4  [81.9, 84.9] *** |
| Male |  | 0.3  [-0.2, 0.7] | 2.7  [2.3, 3.1] *** | 0.7  [0.5, 1.0] *** | 2.1  [1.7, 2.6] *** |  |
| Age group: 50-66 | 2.4  [2.0, 2.7] *** | 1.6  [1.0, 2.1] *** | 2.6  [2.2, 3.1] *** | 5.8  [5.5, 6.1] *** | 4.6  [4.0, 5.2] *** | 3.3  [2.7, 4.0] *** |
| Age group: 67-79 | 1.8  [1.4, 2.2] *** | 2.7  [2.1, 3.3] *** | 3.7  [3.2, 4.2] *** | 8.2  [7.8, 8.6] *** | 5.8  [5.2, 6.4] *** | 5.2  [4.5, 6.0] *** |
| Age group: 80 + | -0.1  [-0.8, 0.6] | 1.4  [0.3, 2.5] * | 2.1  [1.2, 3.0] *** | 8.5  [7.9, 9.2] *** | 3.5  [2.5, 4.5] *** | 3.8  [2.5, 5.1] *** |
| Self-reported long-term condition: One | -1.8  [-2.2, -1.4] *** | -1.2  [-1.8, -0.7] *** | -4.4  [-4.9, -3.9] *** | -0.5  [-0.9, -0.2] ** |  | 0.4  [-0.3, 1.2] |
| Self-reported long-term condition: Two | -3.0  [-3.4, -2.6] *** | -2.8  [-3.4, -2.1] *** | -6.4  [-6.9, -5.8] *** | -1.0  [-1.4, -0.6] *** | -1.9  [-2.4, -1.4] *** | 0.6  [-0.2, 1.4] |
| Self-reported long-term condition: Three or more | -4.1  [-4.6, -3.6] *** | -3.8  [-4.5, -3.2] *** | -7.9  [-8.5, -7.3] *** | -1.1  [-1.6, -0.7] *** | -3.6  [-4.2, -3.0] *** | 0.9  [0.1, 1.8] * |
| Num.Obs. | 58683 | 57167 | 52719 | 58143 | 38221 | 58394 |
| AIC | 502715.3 | 536259.6 | 473452.5 | 493658.9 | 345643.1 | 575785.0 |
| BIC | 502796.1 | 536349.1 | 473541.2 | 493748.6 | 345720.0 | 575865.8 |
| Municipality variance | 11.4 | 63.3 | 11.7 | 24.4 | 13.7 | 144.9 |
| Residual variance | 304.4 | 684.0 | 461.1 | 281.0 | 489.9 | 1103.7 |
| RMSE | 17.41 | 26.09 | 21.42 | 16.72 | 22.07 | 33.14 |

*** p < 0.01, ** p < 0.05, * p<0.1. Confidence interval (CI).
